# Down regulation of the expression of mitochondrial phosphopantetheinyl-proteins in pantothenate kinase-associated neurodegeneration: pathophysiological consequences and therapeutic perspectives

**DOI:** 10.1186/s13023-021-01823-3

**Published:** 2021-05-05

**Authors:** Mónica Álvarez-Córdoba, Marta Talaverón-Rey, Irene Villalón-García, Suleva Povea-Cabello, Juan M. Suárez-Rivero, Alejandra Suárez-Carrillo, Manuel Munuera-Cabeza, Joaquín J. Salas, José A. Sánchez-Alcázar

**Affiliations:** 1 Centro Andaluz de Biología del Desarrollo (CABD), Consejo Superior de Investigaciones Científicas, Universidad Pablo de Olavide, Carretera de Utrera Km 1, 41013 Sevilla, Spain; 2 Centro de Investigación Biomédica en Red: Enfermedades Raras, Instituto de Salud Carlos III, 41013 Sevilla, Spain; 3 Departamento de Bioquímica Y Biología Molecular de Productos Vegetales, Instituto de La Grasa (CSIC), Sevilla, Spain

**Keywords:** Pantothenate kinase, Pantothenate kinase-associated neurodegeneration, Coenzyme A, Mitochondria, Pantothenate, Induced neurons, Acyl carrier protein, 4′-phosphopantetheinylation

## Abstract

**Background:**

Neurodegeneration with brain iron accumulation (NBIA) is a group of genetic neurological disorders frequently associated with iron accumulation in the basal nuclei of the brain characterized by progressive spasticity, dystonia, muscle rigidity, neuropsychiatric symptoms, and retinal degeneration or optic nerve atrophy. Pantothenate kinase-associated neurodegeneration (PKAN) is the most widespread NBIA disorder. It is caused by mutations in the gene of pantothenate kinase 2 (PANK2) which catalyzes the first reaction of coenzyme A (CoA) biosynthesis. Thus, altered PANK2 activity is expected to induce CoA deficiency as well as low levels of essential metabolic intermediates such as 4′-phosphopantetheine which is a necessary cofactor for critical proteins involved in cytosolic and mitochondrial pathways such as fatty acid biosynthesis, mitochondrial respiratory complex I assembly and lysine and tetrahydrofolate metabolism, among other metabolic processes.

**Methods:**

In this manuscript, we examined the effect of PANK2 mutations on the expression levels of proteins with phosphopantetheine cofactors in fibroblast derived from PKAN patients. These proteins include cytosolic acyl carrier protein (ACP), which is integrated within the multifunctional polypeptide chain of the fatty acid synthase involved in cytosolic fatty acid biosynthesis type I (FASI); mitochondrial ACP (mtACP) associated with mitocondrial fatty acid biosynthesis type II (FASII); mitochondrial alpha-aminoadipic semialdehyde synthase (AASS); and 10-formyltetrahydrofolate dehydrogenases (cytosolic, ALD1L1, and mitochondrial, ALD1L2).

**Results:**

In PKAN fibroblasts the expression levels of cytosolic FAS and ALD1L1 were not affected while the expression levels of mtACP, AASS and ALD1L2 were markedly reduced, suggesting that 4′-phosphopantetheinylation of mitochondrial but no cytosolic proteins were markedly affected in PKAN patients. Furthermore, the correction of PANK2 expression levels by treatment with pantothenate in selected mutations with residual enzyme content was able to correct the expression levels of mitochondrial phosphopantetheinyl-proteins and restore the affected pathways. The positive effects of pantothenate in particular mutations were also corroborated in induced neurons obtained by direct reprograming of mutant PANK2 fibroblasts.

**Conclusions:**

Our results suggest that the expression levels of mitochondrial phosphopantetheinyl-proteins are severely reduced in PKAN cells and that in selected mutations pantothenate increases the expression levels of both PANK2 and mitochondrial phosphopantetheinyl-proteins associated with remarkable improvement of cell pathophysiology.

**Supplementary Information:**

The online version contains supplementary material available at 10.1186/s13023-021-01823-3.

## Background

The term Neurodegeneration with Brain Iron Accumulation (NBIA) refers to a group of genetic and progressive neurodegenerative diseases characterized by dystonia, rigidity, and choreoathetosis caused by iron accumulation in certain parts of the brain mainly basal ganglia [1, 2]. Currently, 15 genes have been identified to cause the main clinical entities of NBIA [3]. However, the causative mutation is unknown in around 20% of cases [4].

More than 50% of cases of NBIA are originated by mutations in the gene of pantothenate kinase 2 (PANK2) which encodes an essential enzyme in coenzyme A (CoA) biosynthesis [5]. This clinical subtype is termed pantothenate kinase-associated neurodegeneration (PKAN). The pantothenate kinase gene family includes PANK1a, PANK1b, PANK2 and PANK 3, but only the PANK2, is the gene responsible for PKAN. PANK2 enzyme is localized in mitochondrial intermembrane space and transforms (R)-pantothenate into (R)-4′-phosphopantothenate using ATP.

The enzyme alteration causes coenzyme A deficiency, mitochondria dysfunction and low energy production, intracellular iron accumulation, alterations in cell membranes renewal and impaired protection against oxidative damage, which provokes lipid peroxidation and pathological changes of cell membranes, and eventually cell demise [4, 6]. Altered mitochondrial membrane potential and defective mitochondrial respiration have been demonstrated in PANK2-defective neurons derived from KO mice [7] and in cellular models derived from PKAN patients [8–10]. However, the precise pathological mechanisms involved in PKAN are not completely understood.

Apart of metabolic alterations including impairment of the citric acid cycle, sterol and steroid biosynthesis, heme biosynthesis, amino acid synthesis, and β-oxidation [11], low CoA levels particularly in mitochondria can also affect the 4′-phosphopantetheinylation of essential proteins for mitochondrial function and cell homeostasis [12]. The rationale is that CoA is the supply source for the 4′-phosphopantetheine moiety needed for the posttranslational 4′-phosphopantetheinylation required to activate specific proteins.

Thus, multi-enzyme complexes which sequentially catalyse several reactions are often dependent on the covalent binding of a 4′-phosphopantetheine cofactor to specific proteins. This protein carries metabolic intermediates in the process of different enzymatic reactions. In mammals, the transfer of the 4′-phosphopantetheinyl cofactor from coenzyme A to specific proteins takes place following protein biosynthesis as a post-translational modification [13]. Thus, 4′-phosphopantetheinylation is necessary for the transformation enzymes into their full-active forms [13].

In mammals, 4′-phosphopantetheinylation is required for several enzymes, including acyl carrier protein (ACP) in type I Fatty Acid Synthesis (FAS) and mitochondrial ACP (mtACP) in type II mitochondrial FAS, α-Aminoadipate semialdehyde synthase (AASS) in lysine metabolism and 10-formyltetrahydrofolate dehydrogenase (10-FTHFDH) with two isoforms (Cytosolic 10-FTHFDH or ALDH1L1 and mitocondrial 10-FTHFDH or ALDH1L2) involved in folate metabolism [13]. Surprisingly, mammals only encode one unique phosphopantetheinyl transferase (PPTase) in their genome which is called L-aminoadipate-semialdehyde dehydrogenase-phosphopantetheinyl transferase (AASDHPPT) [14]. The enzyme catalyzes the hydrolysis of coenzyme A to 3′,5′-adenosine diphosphate and 4′-phosphopantetheine, and the transfer of the 4′-phosphopantetheinyl moiety to a serine residue at the active site of the particular proteins.

The mammalian 10-FTHFDH requires a 4′-phosphopantetheine cofactor for catalysis [15]. This enzyme is phosphopantetheinylated by AASDHPPT, and siRNA silencing of this enzyme completely blocks the post-translational modification of 10-FTHFDH. A mitochondrial homolog of 10-FTHFDH was found to be activated by the same PPTase [16]. The human PPTase AASDHPPT acts on several of *apo*-proteins [13], suggesting that is not specific for particular proteins. Recently, the human PPTase has been crystallized allowing a better knowledge of its molecular mechanism [17].

The mitochondrial localization of PANK2 and the regulation of PANK2 activity by species of acyl CoA may have potential importance for the presence of a independent biosynthesis pathway of fatty acids in type II mitochondrial FAS [18]. The fatty acid synthase complex uses acetyl and malonyl CoA in addition to acyl carrier protein (ACP), which depends on a phosphopantetheine cofactor for its function as an acyl carrier. Although the cytosolic ACP and fatty acid synthetase complex have been described as the main proteins participating in fatty acid synthesis, a mtACP protein different from cytosolic ACP, and containing a phosphopantetheine prosthetic group, has also been identified [19, 20]. The specific meaning for a separate pathway for fatty acid synthesis in the mitochondrial compartment is not completely understood, although several findings show that it may be essential for phospholipid metabolism in mitochondrial membranes [21, 22].

Recently, it has been demonstrated that CoA-dependent activation of mitochondrial acyl carrier protein (mtACP) is a possible process linking several neurodegenerative diseases such as PKAN, CoPAN (CoA synthase protein-associated neurodegeneration), MePAN (Mitochondrial enoyl CoA reductase protein-associated neurodegeneration), and PDH-E2 (pyruvate dehydrogenase-E2) deficiency which share key phenotypic features but harbor defects in distinct metabolic processes [12]. Specific severe damage to the globus pallidus, one of the nuclei that make up the basal ganglia, occurs in these hereditary neurodegenerative disorders, which are caused by defects in CoA biosynthesis (PKAN, CoPAN), protein lipoylation (MePAN), and pyruvate dehydrogenase activity (PDH-E2 deficiency). The authors propose that CoA-dependent activation of mtACP is a possible event connecting these clinical and molecular entities through its effect on PDH activity.

In this work using cellular models derived from PKAN patients, we examine the hypothesis that CoA deficiency caused by PANK2 mutations may affect the expression levels and activity of key mitochondrial proteins harboring a 4′-phosphopantetheiny cofactor such as mtACP, ALDH1L2 or AASS. The pathophysiological and therapeutic consequences of these alterations are also discussed.

## Results

### Expression levels of mitochondrial 4′-phosphopantetheinyl proteins are markedly reduced in fibroblasts derived from PKAN patients

**Fig. 1:**
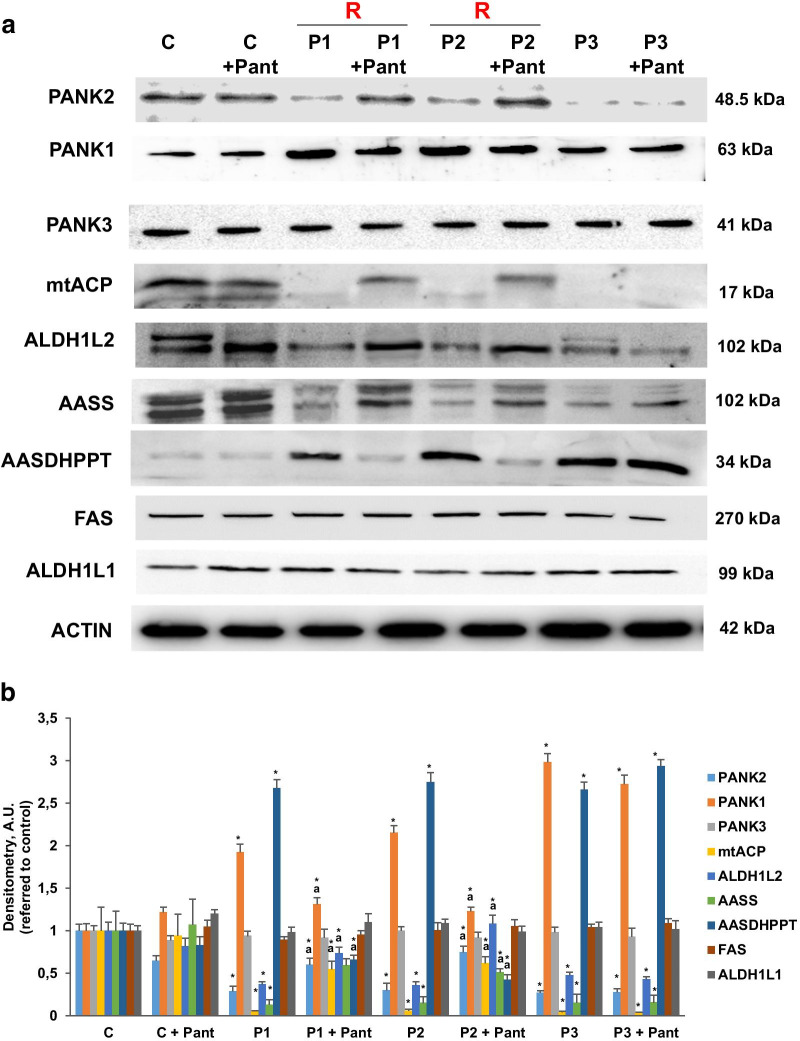
Expression levels of 4′-phosphopantetheinyl proteins in mutant PANK2 fibroblasts. **a** Immunoblotting analysis of cellular extracts from Control (C) and patients P1, P2 and P3 fibroblasts. Cells were treated with 500 μM pantothenate for 20 days. Protein extracts (50 μg) were separated on a SDS polyacrylamide gel and immunostained with antibodies against PANK1, PANK2, PANK3 and the 4′-phosphopantetheinyl proteins cytosolic FAS and ALD1L1 and mitochondrial mt-ACP, AASS and ALD1L2. **b** Densitometry of the Western blotting. Data represent the mean ± SD of three separate experiments. *p < 0.01 between PKAN patients and controls; ^a^*p* < 0.01 between untreated and treated fibroblasts. A.U., arbitrary units. R = responder fibroblasts

In a previous work, we analyzed PANK2 expression levels in fibroblast cell lines derived from three PKAN patients and three healthy subjects [8]. Two PKAN patients, P1 and P2, harbouring compound heterozygous mutations and decreased PANK2 expression levels, while patient P3 carried a homozygous frame shift mutation that results in the complete lack of PANK2 expression [8]. As shown by Western-blot analysis in Fig. 1a, PANK2 expression levels were markedly reduced in patients P1 and P2 and practically absent in P3 while normal expression levels were present in control fibroblasts. As a compensatory mechanism for PANK2 deficiency, expression levels of PANK1 were significantly increased in all three PANK2 mutant fibroblasts. However, expression levels of PANK3 were not altered.

Interestingly, supplementation with pantothenate (500 µM), the substrate of the PANK2 enzyme, was able to stabilize the expression levels of the mutant enzyme in selected patients, P1 and P2, but not in P3 fibroblasts with a frame shift mutation (Fig. 1a, b) [8]. To further characterize the pathological consequences of CoA deficiency in PANK2 mutations, we then examined the expression of cytosolic and mitochondrial proteins carrying 4′-phosphopantetheine cofactors, an intermediate metabolite in the CoA biosynthesis pathway. Expression levels of mitochondrial 4′-phosphopantetheinyl proteins such as mtACP, AASS and ALD1L2 were markedly reduced in PANK 2 fibroblasts. However, the expression levels of cytosolic FAS, which also contains an ACP domain containing phosphopantetheine, and ALD1L1, the cytosolic counterpart of ALD1L2, were not affected. These results suggest that the mitochondrial, but not the cytosolic, phosphopantetheinyl-proteins was severely affected in PKAN. Interestingly, pantothenate treatment significantly restored the expression levels of 4′-phosphopantetheinyl proteins in responder PKAN fibroblasts, P1 and P2, but not in non-responder P3 fibroblasts (Fig. 1a, b).

**Fig. 2:**
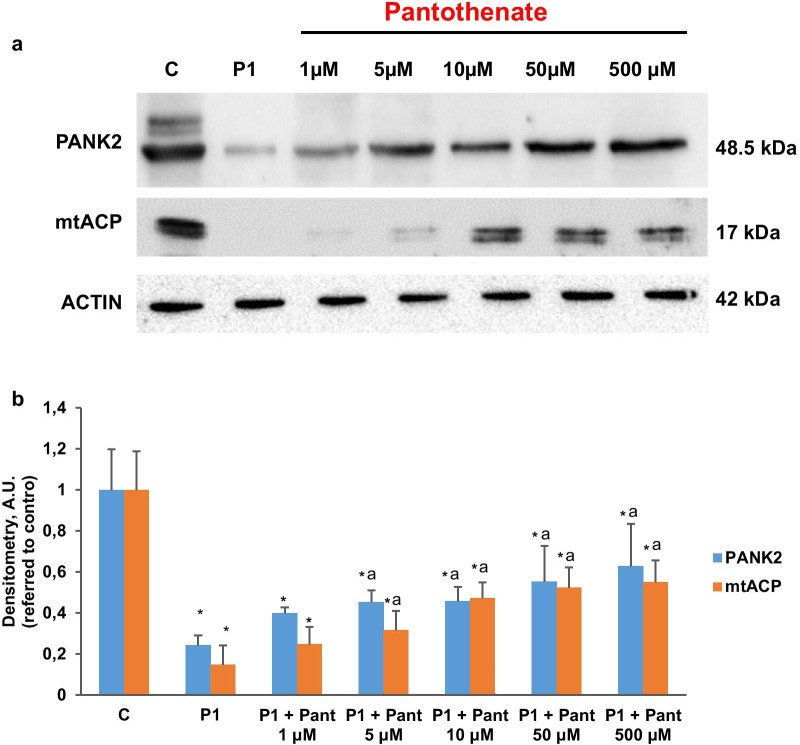
Dose–effect of pantothenate treatment on PANK2 and mt-ACP expression levels. **a** Control (C1) and PKAN fibroblasts (P1, P2 and P3) were treated with increasing concentrations of pantothenate (1, 5, 10, 50, 500 μM) for 20 days. Protein extracts (50 μg) were separated on a SDS polyacrylamide gel and immunostained with antibodies against PANK2 and mt-ACP. **b** Densitometry of the Western blotting. Data represent the mean ± SD of three separate experiments. *p < 0.01 between PKAN patients and controls; ^a^*p* < 0.01 between untreated and treated fibroblasts. A.U., arbitrary units

**Fig. 3:**
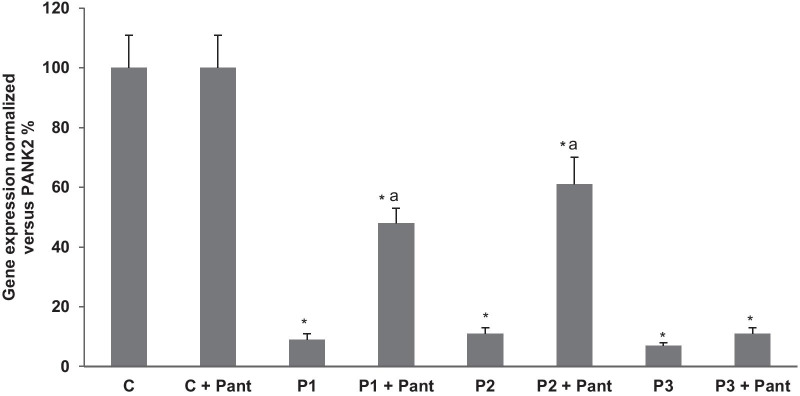
Effect of pantothenate treatment on PANK2 transcripts. Control (C) and patients P1, P2 and P3 fibroblasts were treated with 500 μM pantothenate for 20 days. PANK2 transcripts were quantified by qPCR as described in Material and Methods. Data represent the mean ± SD of three separate experiments. *p < 0.01 between PKAN patients and controls; ^a^*p* < 0.01 between untreated and treated fibroblasts. A.U., arbitrary units

To compensate the low mitochondrial CoA levels in PKAN cells [8], the expression levels of AASDHPPT, the enzyme responsible of the transfer of phosphopantetheine from CoA to particular proteins, were markedly increase in all PKAN fibroblasts. As expected, pantothenate treatment corrected the expression levels of AASDHPPT in responder mutations (P1 and P2) but not in fibroblast harbouring bi-allelic mutations encoding truncated PANK2 proteins (P3). The positive effect of pantothenate on PANK2 and mtACP expression levels was dose dependent in responder mutant fibroblasts (Fig. 2a, b). Furthermore, the favourable effect of pantothenate on PANK2 protein expression levels was associated with an increase in the steady-state levels of PANK2 transcripts (Fig. 3) suggesting that pantothenate was able to up-regulate PANK2 gene expression or transcript stabilization.

### Mitochondrial protein lipoylation was reduced in PKAN fibroblasts

Next, we focused in the pathological alterations potentially induced by mtACP deficiency. Thus, as mtACP is essential for lipoic acid synthesis by mitochondrial FAS II [23], we explored protein lipoylation in control and PKAN fibroblasts. Lipoic acid is a cofactor central to cellular metabolism [24, 25]. As a lysine posttranslational modification on particular components of enzymatic complexes, this functional group is required for the activities of these multimeric complexes [26, 27]. For example, the pyruvate dehydrogenase (PDH) and alpha-ketoglutarate (KDH) complexes regulate carbon entry points into the central metabolic pathway of the tricarboxylic acid cycle (TCA) cycle [28]. On both complexes, lipoylation is critical for proper enzyme function, and deficiency of this modification inhibits their activities.

**Fig. 4:**
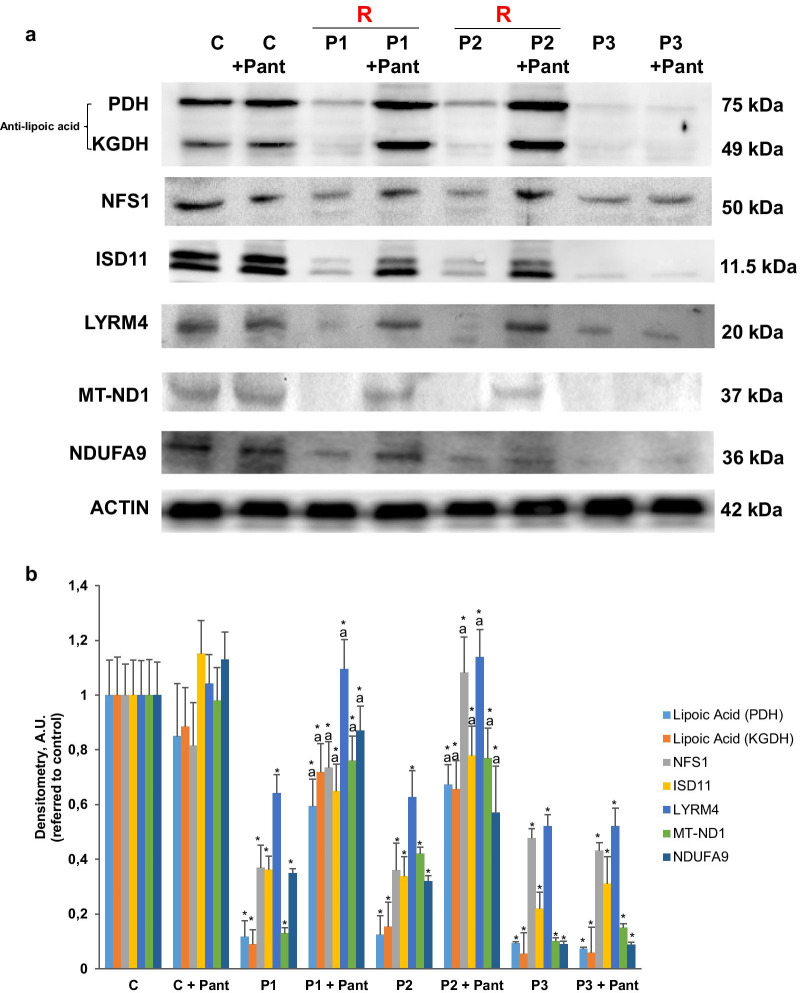
Expression levels of lipoyl proteins, Fe-S cluster complex proteins and mitochondrial complex I subunits in mutant PANK2 fibroblasts. Control (C) and PKAN fibroblasts (P1 and P3) were treated with 500 μM pantothenate for 20 days. **a** Immunoblotting analysis of cellular extracts from Control (C) and patients P1, P2 and P3 fibroblasts. Protein extracts (50 μg) were separated on a SDS polyacrylamide gel and immunostained with antibodies against lipoic acid, Fe-S cluster complex proteins NFS1, ISD11 and LYRM4 and mitochondrial complex I subunits MT-ND1 and NDUFA9. **b** Densitometry of the Western blotting. Data represent the mean ± SD of three separate experiments. *p < 0.01 between PKAN patients and controls; ^a^*p* < 0.01 between untreated and treated cells. A.U., arbitrary units. R = responder fibroblasts

**Fig. 5:**
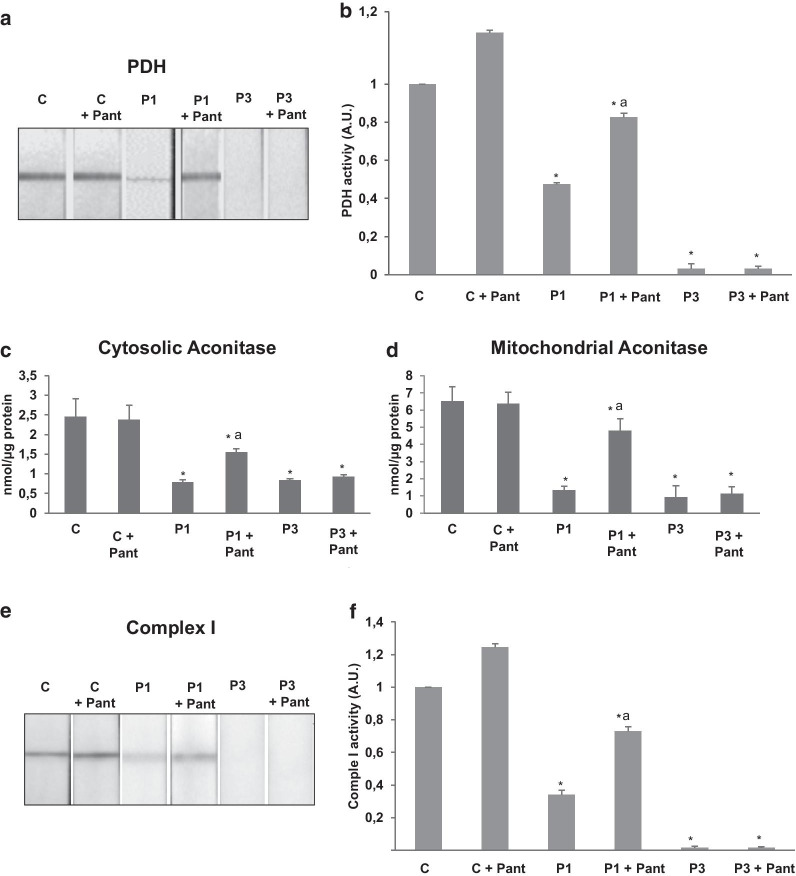
Reduced PDH, aconitase activities and complex I activity in mutant PANK2 fibroblasts. Control (C), P1 and P3 fibroblasts were treated with 500 μM pantothenate for 20 days. **a**, **b** PDH activity in whole cellular extracts was determined as described in Material and Methods. **c**, **d** Cytosolic and mitochondrial aconitase activity in whole cellular extracts was determined as described in Material and Methods. **e**, **f** Mitochondrial complex I activity in whole cellular extracts was determined as described in Material and Methods. Data represent the mean ± SD of three separate experiments. *p < 0.01 between PKAN patients and controls; ^a^*p* < 0.01 between untreated and treated cells

As in shown in Fig. 4a, b, PDH and KDH lipoylation were drastically reduced in PKAN fibroblasts. Accordingly, the enzymatic activity of PDH was markedly reduced in PKAN fibroblast (Fig. 5a). Interestingly, pantothenate supplementation was able to increase both mitochondrial protein lipoylation (Fig. 4a, b) and restore partially PDH activity (Fig. 5a, b) in responder mutant PANK2 fibroblasts.

### Expression of iron-sulfur cluster biosynthesis proteins were also affected in PKAN

As ACP also participates in Fe/S cluster biosynthesis [29], we then explored the expression levels of several proteins participating in the mitochondrial Fe/S cluster synthesis complex. As displayed in Fig. 4a, b, expression levels of NFS1 (NFS1 cysteine desulfurase), ISD11(Iron-sulfur protein biogenesis, desulfurase-interacting protein 11) and LYRM4 (LYR Motif Containing 4) were dramatically reduced in PANK2 mutant fibroblast, suggesting that ACP deficiency causes a disassembly of the Fe/S cluster synthesis complex and, as a consequence a deficiency of Fe-S cofactor biosynthesis. To confirm the hypothesis we next examined cytosol and mitochondrial aconitase activity, an enzyme that dependent of Fe-S prostetic groups. Both cytosolic and mitochondrial aconitase activities were significantly reduced in PKAN mutant fibroblasts. As expected, pantothenate treatment was able to restore their activities to control levels in responder mutant cells with residual PANK2 enzyme expression (Fig. 5c, d).

### Expression levels of respiratory complex I proteins were reduced in PKAN fibroblasts

As mtACP is also critically involved in the assembly of mitochondrial respiratory complex I [30], we next evaluated both the expression levels of two subunits of complex I, MT-ND1 and NDUFA9, and complex I activity in control and PANK2 mutant fibroblasts.

Expression levels of MT-ND1 and NDUFA9 were markedly reduced in PKAN fibroblasts suggesting complex I disorganization (Fig. 4a, b). In agreement with these results the activity of complex I was significantly reduced in mutant fibroblasts (Fig. 5e, f). The restoration of PANK2 expression levels by pantothenate was also able to restore the expression levels of complex I subunits (Fig. 4a, b) and complex I enzymatic activity (Fig. 5e, f).

### Generation of induced neurons from control and PANK2 mutant fibroblasts

To further demonstrate the beneficial effect of pantothenate in specific PANK2 mutations, control and patient responder fibroblasts were transdifferentiated to induced neurons by direct reprograming. Thus, control and PANK2 mutant fibroblasts were infected with lentiviral vectors expressing proneural genes Ascl1 and Blc2 and promoting the knock down of the REST complex [31]. After transdifferentiation, cells manifested a typical neuron-like morphology and showed positive immunoreactivity against two neuron-specific proteins, Tau and MAP2 (microtubule associated protein 2). In contrast, undifferentiated fibroblasts did not show Tau or MAP2 staining. Positive cells for Tau and MAP2 were used to evaluate neuronal conversion efficiency, which was approximately 50% in control cells and 20% in PANK2 mutant cells (Additional file 1: Fig. S1a). Neuronal purity was almost 35% in control cells and 55% in PANK2 mutant cells (Additional file 1: Fig. S1b).

**Fig. 6:**
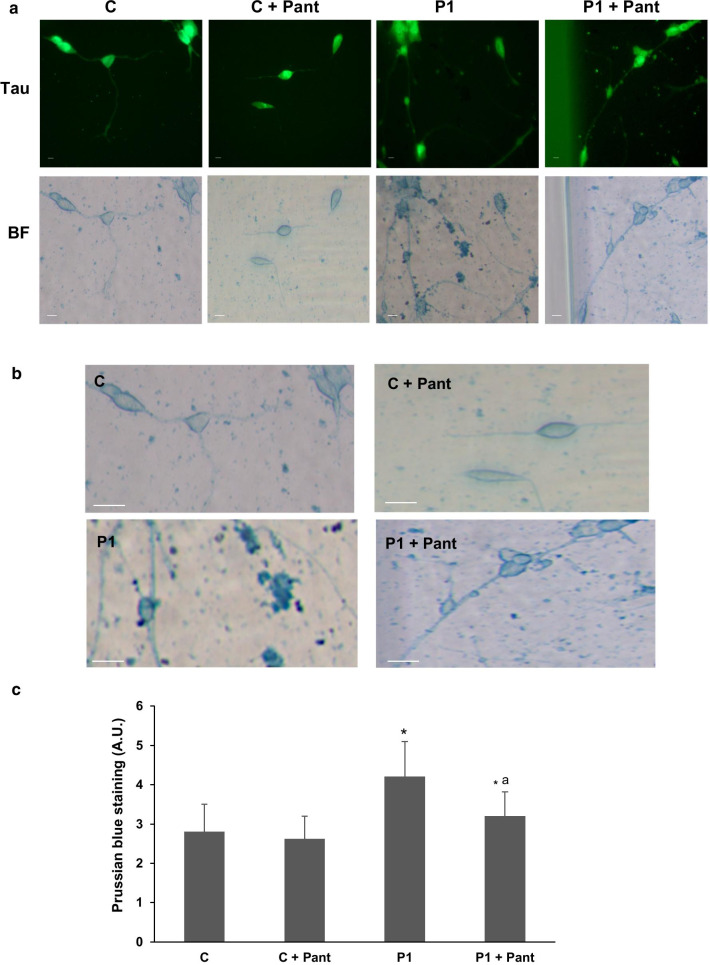
Mutant PANK2 induced neurons show iron accumulation. **a** Tau positive immunostaining and bright-field (BF) of Prussian Blue staining images of control (C) and P1 induced neurons after 500 μM pantothenate treatment during 20 days. Scale bar = 15 μm. **b** Representative bright-field images at higher magnification of Control (C) and P1 induced neurons after Prussian Blue staining. Scale bar = 15 μm. **c** Quantification of Prussian Blue staining. Data represent the mean ± SD of three separate experiments. *p < 0.01 between PKAN patients and controls; ^a^*p* < 0.01 between untreated and treated cells

Next, the beneficial effect of pantothenate in mutant PANK2 induced neurons derived from P1 fibroblasts, which respond positively to pantothenate supplementation, was evaluated by examining iron accumulation using Prussian Blue staining. PANK2 mutant induced neurons showed increased Prussian Blue staining indicating iron accumulation (Fig. 6a–c). As expected, iron accumulation was eliminated after 500 µM pantothenate treatment (Fig. 6a–c).

**Fig. 7:**
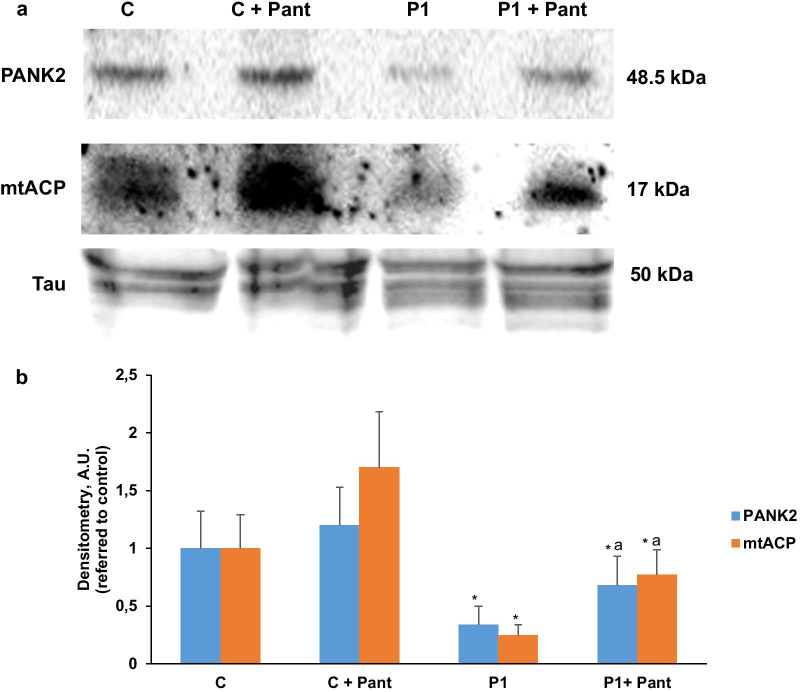
Pantothenate corrected PANK2 and mtACP deficiency in mutant PANK2 induced neurons. **a** Immunoblotting analysis of cellular extracts from Control (C) and patient P1 induced neurons treated with 500 μM pantothenate during 20 days. Protein extracts (5 μg) were separated on a SDS polyacrylamide gel and immunostained with antibodies against PANK2 and mtACP. Tau expression levels in induced neurons were used as loading control. **b** Densitometry of the Western blotting. Data represent the mean ± SD of three separate experiments. *p < 0.01 between Control and mutant PKAN induced neurons. ^a^*p* < 0.01 between untreated and treated induced neurons. A.U., arbitrary units

To corroborate the beneficial effect of pantothenate treatment, PANK2 and ACP expression levels were addressed in induced neurons. As shown in Fig. 7a, b, pantothenate treatment was able to correct the expression levels of both proteins in mutant PANK2 induced neurons.

## Discussion

Our results demonstrate that mitochondrial 4′-phosphopantetheinyl-proteins are severely reduced in mutant PANK2 cells and that pantothenate treatment in selected mutations increases the expression levels of both PANK2 and mitochondrial phosphopantetheinyl-proteins. This result is predicted because mitochondrial CoA levels, the source for the 4′-phosphopantetheinylation in this organelle, are low in PANK2 mutations [8]. On the contrary, the increase of PANK2 levels by pantothenate treatment may increase mitochondria CoA levels in responder mutations [8] and, as a consequence, may correct at least partially mitochondrial 4′-phosphopantetheinyl-proteins levels.

Consistent with a markedly decrease of PANK2 activity and mitochondrial CoA levels, as previously demonstrated by our group [8], the expression levels of 4′-phosphopantetheinyl proteins such as mtACP, ALDH1L2 and AASS were dramatically reduced in mutant PANK2 cells. In contrast, the expression levels of AASDHPPT which catalyzes the transfer the 4′-phosphopantetheine moiety from coenzyme A to target proteins, were increased, presumably as a compensatory mechanism to overcome the low mitochondrial CoA levels and the 4′-phosphopantetheinylation defect. Our data suggest that the down-regulation of phosphopantetheinyl- proteins in PANK2 mutant fibroblast was confined to mitochondria because the expression levels of cytosolic 4′-phosphopantetheinyl proteins such as ALDH1L1 and FAS were normal.

In agreement with mtACP deficiency, which impairs lipoic acid biosynthesis in mitochondria, we also show a specific influence of impaired CoA biosynthesis on lipoylated protein levels. Thus, PDH lipoylation and activity were severely reduced in PKAN cells. The activity of other enzyme complexes such as αKGDH that are regulated by lipoylation [32] are presumably also affected because the levels of lipoylated αKGDH are dramatically reduced (Fig. 4a, b).

Furthermore, mt-ACP deficiency may also affect several metabolic pathways because mtACP serves to numerous and crucial mitochondrial functions. Indeed, NDUFAB1, the human ortholog of mtACP, is a subunit (and required for its assembly) of mitochondrial respiratory complex I [33, 34] and it is also involved in iron-sulfur biogenesis [35]. Thus, the prediction would be that under conditions of mtACP deficiency, complex I activity and iron-sulfur cluster formation would be also decreased, which is in agreement with the results obtained by Jeong et al. in a mouse model of PKAN [36] and the findings of Lambrechts et al. in Drosophila models of CoA deficiency [12]. In the mouse model of PKAN they demonstrated the presence of a specific alterations in the globus pallidus including impaired complex I activity, reduced activity of PDH and Fe-S dependent enzymes. All these findings are consistent with a primary defect in 4′-phosphopantetheinylated mtACP [36]. Likewise, in the Drosophila model under conditions of CoA deficiency, mtACP levels were decreased associated with reduced protein lipoylation and PDH activity [12].

Furthermore, it was recently shown in eukaryotic cells that mtACP is involved in iron-sulfur cluster biogenesis and stability, indicating an essential role for the 4′- phosphopantetheinyl modification of mitochondrial proteins [37] In *Saccharomyces cerevisiae*, loss of mtACP leads to reduced iron-sulfur cluster formation, inactivation of Fe-S cluster-dependent enzymes such as aconitase, and activation of iron-responsive factors Aft1 and Aft2 [35]. Consistently, decreased Fe-S cluster levels result in mitochondrial iron accumulation [38]. Abnormal iron metabolism and decreased aconitase activity are characteristic features of mutant PANK2 fibroblasts, as well as induced pluripotent stem cells-derived neurons [9, 10].

Our observations in cellular models derived from PKAN patients corroborated all these predictions and observations. Thus, mutant PANK2 fibroblasts showed marked reductions in both mitochondrial complex I activity as well as aconitase activity, which depends on Fe-S center biogenesis.

It is interesting to note that although the altered PANK2 activity causes perturbations in the expression of iron-sulfur cluster biosynthesis proteins such as NFS1, ISD11 and LYRM4, the clinical and neuroimaging findings in patients with PKAN and related disorders are quite different from primary disorders of Fe–S cluster biogenesis (globus pallidus involvement in PKAN *versus* the preferential leukoencephalopathy pattern seen in FE-S cluster biogenesis disorders) [12, 39]. This heterogeneity of clinical manifestations although needs a better clarification can be ascribed to the fact that PANK2 defect (affecting ACP expression levels) also affects mitochondrial phospholipids biosynthesis as well as lysine and folate metabolism among others essential mitochondrial pathways [13]. Several critical questions still remain such as: Why do PANK2 deficiency and some defects (but not all) of Fe–S cluster biogenesis cause iron overload? Why is iron preferentially accumulated in basal ganglia in PKAN patients?

Our observations together with the findings of other authors [12] have implications for potential therapeutic approaches for PKAN. Thus, replacement with lipoic acid [40] and/or PDH boosting agents such as dichloroacetate (DCA) [41] or thiamine [42] may help as complementary therapies in PKAN. However, the alteration of iron metabolism or other dysfunctions emerging from downstream of mtACP activity such as of aconitase and complex I activities may not benefit from this approach [12]. Nevertheless, in responder mutations the best option would be pantothenate treatment that is able to increase PANK expression, correct CoA levels in mitochondria [8], and normalize the expression levels of mtACP and other phosphopantetheinyl proteins in cellular models of PKAN. For those patients with pantothenate non-responder mutations this strategy is not feasible and the solution it would be to bypass the PANK2 defect with 4′-phosphopantetheine or similar molecules [12].

Pantothenate is the substrate of the defective enzyme PANK2. The existence of residual enzyme activity in some individuals with PKAN has suggested the possibility of treatment using high-dose pantothenate [43], given that it has no known toxicity in humans. However, the efficacy of pantothenate supplementation in ameliorating symptoms has not been systematically demonstrated; some individuals have reported improvement in their symptoms under pantothenate treatment [1]. According to our results is critical the identification of patients that can respond positively to pantothenate by examining the cellular response to the treatment. Likewise, from a clinical point of view it would be important to find out the optimal concentration of pantothenate capable of correcting pathophysiological alterations in the cell models of particular mutations. For this reason, precision personalised medicine approach in PKAN together with the pharmacokinetics data of different pantothenate formulations can provide a useful information for making appropriate therapeutic decisions [44].

In our work, the positive effect of pantothenate has been also proved in induced neurons derived from PKAN patients. Thus, pantothenate was able to eliminate iron accumulation and significantly increase the expression of PANK2 and mtACP in responder mutations confirming previous findings of our group [8].

For future investigations, it could be also interesting to explore the positive effect of pantothenate on other pathological alterations present in PKAN such as accantocytosis which is due to alterations of plasma membrane proteins and/or lipids in erythrocytes [45].

## Conclusion

We show that impaired CoA homeostasis due to PANK2 mutations leads to decreased expression levels of essential mitochondrial proteins such as mtACP which participates in lipoic acid biosynthesis, and consequently affects protein lipoylation and activity of lipoylated proteins including PDH. Furthermore, mtACP deficiency was associated to reduce mitochondrial complex I activity and down-regulation of proteins forming the Fe/S cluster synthesis complex.

These findings support the hypothesis that PANK2 mutations dramatically alter mitochondrial function affecting the expression levels of mitochondrial phosphopantetheinyl-proteins. Therefore, expression levels of these proteins can be excellent biomarkers to address disease severity and effectiveness of potential treatments. Thus, in responder mutations, pantothenate can rescue PANK2 and all pathological alterations including mtACP levels, PDH and complex I activity, and the expression of Fe–S cluster proteins.

Our results suggest that alterations in mitochondrial metabolism such as lipoic acid synthesis, complex I assembly and Fe–S cluster biogenesis may underlie the neurodegenerative process in PKAN.

## Methods

### Reagents

Monoclonal Anti-actin antibody, anti-fatty acid synthase (FAS), Prussian Blue, sodium pantothenate and trypsin were purchased from Sigma Chemical Co. (St. Louis, MO). Anti-mitochondrial acyl carrier protein (mtACP), anti-aminoadipate semialdehyde dehydrogenase phosphopantetheinyl transferase (AASDHPPT), anti-NADH dehydrogenase [ubiquinone] 1 alpha subcomplex subunit 9 (NDUFA9), anti-NADH-ubiquinone oxidoreductase chain 1 (MTND1), DAPI and Hoechst 3342, were purchased from Invitrogen/Molecular Probes (Eugene, OR). NFS1 antibodies were purchased from Santa Cruz Biotechnology (Santa Cruz, CA). Anti-PANK2, complex 1 activity kit, PDH activity kit and aconitase activity kit were purchased from Abcam (Cambridge, UK). Anti-mitochondrial 10-formyltetrahydrofolate dehydrogenase (ALDH1L2), anti-cytosolic 10-formyltetrahydrofolate dehydrogenase (ALDH1L1), anti-alpha-aminoadipic semialdehyde synthase, mitochondrial (AASS), anti-LYRM4, anti-Tau clone HT7, anti-ISD11, anti-PANK1 and anti-PANK3 were purchased from Thermo-Fisher (Waltham, MA). Anti-lipoic acid was acquired from Merck (Darmstadt, Germany). A cocktail of protease inhibitors (complete cocktail) was purchased from Boehringer Mannheim (Indianapolis, IN). The Immun Star HRP substrate kit was from Bio-Rad Laboratories Inc. (Hercules, CA).

### Ethical statements

Approval of the ethical committee of the Hospital Universitario Virgen Macarena y Virgen de Rocío de Sevilla (Spain) was obtained, according to the principles of the Declaration of Helsinki and all the International Conferences on Harmonization and Good Clinical Practice Guidelines.

### Cell culture

We used primary skin fibroblasts from three unaffected subjects (control 1, 2 and 3, two adults and one neonatal) purchased from ATCC and three patients from the Movement Disorder Unit of Hospital Universitario Virgen del Rocío, Sevilla, Spain, and from the Movement Disorders Bio-Bank available at the Neurogenetics Unit of the Neurological Institute ‘Carlo Besta’ (INCB), Milan, Italy. One patient (P1) is compound heterozygous carrier of changes c.[747dup] that causes a frameshift (p.Arg249Profs) mutation triggering a premature stop codon and c.[1475C>T] (p.Ala492Gly) that causes a missense mutation which is predicted to be damaging by prediction tools such as PolyPhen2 [46]. The second patient (P2) is compound heterozygous carrier of changes in position c.[240_241del] and c.[650C>T] (p.Asp217Gly) which have been previously described [47]. The third patient (P3) P3 carries a homozygous mutation c.[1259delG] causing a frameshift p.[Gly420Valfs*30] mutation [48]. The reference sequence used for the PANK2 mutations was NM_153638. Control values represent means ± SD for three control fibroblast cell lines. Fibroblasts were grown in DMEM (Sigma) supplemented with 10% FBS (Sigma), 100 mg/ml streptomycin, 100 U/ml penicillin and 4 mM l-glutamine (Sigma). All the experiments were performed with fibroblasts cell cultures with a passage number < 10.

### Immunoblotting

Western blotting was performed using standard Methods described in previous manuscripts of the research group [8]. After protein transfer, membranes were incubated with various primary antibodies diluted 1:1000, and then with the corresponding secondary antibody coupled to horseradish peroxidase at a 1:10,000 dilution. Specific protein complexes were identified using the Immun Star HRP substrate kit (Biorad Laboratories Inc., Hercules, CA, USA).

Protein loading was assessed by Ponceau staining and actin expression levels. If the molecular weight of proteins did not interfere, membranes were re-probed with different antibodies. In the case of proteins with different molecular weights, membranes were cut and incubated with specific antibodies.

### Immunofluorescence microscopy

For immunofluorescence studies, we followed a protocol previously described by our research group [49]. Cells were grown on 1 mm width (Goldseal No. 1) glass coverslips for 24–48 h in DMEM containing 20% FBS. Cells were rinsed once with PBS, fixed in 3.8% paraformaldehyde for 5 min at room temperature, and permeabilized in 0.1% saponin for 5 min. For immunostaining, glass coverslips were incubated with primary antibodies diluted 1:100 in PBS, 1–2 h at 37 °C in a humidified chamber. Unbound antibodies were removed by washing the coverslips with PBS (three times, 5 min). The secondary antibody, a FITC-labelled goat anti-mouse antibody or a tetramethyl rhodamine goat anti-rabbit (Molecular Probes), diluted 1:100 in PBS, were added and incubated for 1 h 37 °C. Coverslips were then rinsed with PBS for 3 min, incubated for 1 min with PBS containing Hoechst 33,342 (1 µg/ml) and washed with PBS (three 5 min washes). Finally, the coverslips were mounted onto microscope slides using Vectashield Mounting Medium (Vector Laboratories, Burlingame, CA, USA) and analyzed using an upright fluorescence microscope (Leica DMRE, Leica Microsystems GmbH, Wetzlar, Germany). Colocalization studies were performed using a DeltaVision system (Applied Precision; Issaquah, WA) with an Olympus IX-71 microscope (Olympus Corporation, Shinjuku, Tokyo, Japan).

### Real-time quantitative PCR

Expression of PANK2 gene in fibroblasts was analysed by real time quantitative PCR using mRNA extracts. mRNA was extracted by using standard methods and SYBR Green protocol as a method designed to detect accurate quantification of gene expression and RT-PCR reactions. PANK2 primers used 5′ TTCCCACTCATGACATGCCT-3′ (Forward primer) and 5′-GTGACCGTCCATTGAATCCG-3′ (Reverse primer) amplifying a sequence of 215 nucleotides. Actin was used as a housekeeping control gene and the primers were 5′-AGAGCTACGAGCTGCCTGAC-3′ (Forward primer) and 3′-AGCACTGTGTTGGCGTACAG-5′ (reverse primer).

### Complex I activity

Complex I activity in whole cells was measured using the Complex I Enzyme Activity Dipstick Assay Kit (ab109720, ABCAM, Cambridge, MA, USA) according to manufacturer’s instructions. Three biological replicates were used per measurement. Results are expressed as enzyme activity respect to control. The signal intensity was analyzed by a Molecular Imager ChemiDoc XRS + System (Bio-Rad Laboratories Inc., USA).

### PDH activity

PDH complex activity in whole cells was measured using the Pyruvate dehydrogenase (PDH) Enzyme Activity Dipstick Assay Kit (ab109882, ABCAM, Cambridge, MA, USA) according to manufacturer’s instructions. Three biological replicates were used per measurement. Results are expressed as enzyme activity respect to control. The signal intensity was analyzed by a Molecular Imager ChemiDoc XRS + System (Bio-Rad Laboratories Inc., USA).

### Cell fractionation

Cells were harvested and homogenized using a fractionation buffer containing 250 mM sucrose, 10 mM Tris, 1 mM EDTA and proteases inhibitors cocktail, pH 7.4. Cell suspension was passed through a 25-gauge needle 10 times using a 1 mL syringe. Next, nuclei and intact cells were removed by centrifugation at 1500 g for 20 min. The supernatant containing intact mitochondria was transferred into a new tube and centrifuged at 12,000*g* for 10 min (“mitochondria fraction”. Supernatant (“cytosolic fraction”) was transferred into another new tube. Cytosolic fractions were concentrated using Centricon YM-10 devices (Millipore) according to the manufacturer’s instructions.

### Aconitase activity mitochondria and cytosolic

Cytosolic and mitochondria fraction were used for aconitase activity using the Aconitase Activity Assay Kit (ab83459, ABCAM, Cambridge, MA, USA) according to manufacturer’s instructions. Three biological replicates were used per measurement. Results are expressed as enzyme activity respect to control. Absorbance at 450 nm was measured using a POLARstar Omega Microplate Reader.

### Generation of induced neurons from fibroblasts by direct reprogramming

Neurons were generated from patient and control fibroblasts by direct reprogramming as previously described by Drouin-Ouellet et al. [15, 50]. Controls and patients-derived fibroblasts were plated onto 0.1% gelatin-coated 24-well plates or µ-Slide 4 Well Ibidi plates (2.8e4 cells/cm^2^). The day after, dermal fibroblasts were transduced with one-single lentiviral vector containing neural lineage-specific transcription factors (Acsl1 and Brn2) and two shRNA against the RE1-silencing transcription factor (REST) complex, generated as previously described [51]. The plasmid was a gift from Dr. Malin Parmar (Developmental and Regenerative Neurobiology, Lund University, Sweden). Transduction was performed at a multiplicity of infection (MOI) of 30. The day after, the cells were switched into fresh fibroblast medium and after a further 48 h, the medium was replaced with neural differentiation medium (NDiff227; Takara-Clontech) supplemented with neural growth factors and small molecules at the concentrations previously described [15]: LM-22A4 (2 µM, R&D Systems), GDNF (2 ng/ml, R&D Systems), NT3 (10 ng/ml, R&D Systems), db-cAMP (0.5 mM, Sigma), CHIR99021 (2 µM, Sigma), SB-431542 (10 µM, R&D Systems), noggin (50 ng/ml, R&D Systems), LDN-193189 (0.5 M, Sigma), valproic acid sodium salt (VPA; 1 mM, Sigma). The medium was changed every 2–3 days for a further 10 days. The medium was replaced with neuronal medium supplemented with only growth factors until the end of the conversion. Neuronal cells were identified by the expression of Tau or MAP2. DAPI + and Tau + /MAP2 + cells were considered induced neurons. Conversion efficiency was calculated as the number of Tau + cells over the total number of fibroblasts seeded for conversion. Neuronal purity was calculated as the number of Tau + cells over the total cells in the plate after reprogramming.

For neuronal enrichment for Western blot analysis, eighteen days post-infection, neurons were detached and seeded in 24 well culture plates coated using polyornithine (15 μg/ml), fibronectin (0.5 ng/μl) and laminin (5 μg/ml) to increase the purity of the iNs culture up to 95% without the need of further purification steps.

### Statistical analyses

Statical analysis was routinely performed as formerly described by our research group [52]. We used non-parametric statistics that do not have any distributional assumption in cases when number of events was small (n < 30) [53]. In these cases, multiple groups were compared using a Kruskal–Wallis test. In case of only two groups, they were compared using a Mann–Whitney test. In cases when number of events was higher (n > 30), we applied parametric tests. In these cases, multiple groups were compared using a one-way ANOVA. Bonferroni post-hoc testing was employed after ANOVA for testing for significant differences between groups. In case of only two groups, they were compared using a Student’s t-test with a Welch’s correction. Statistical analyses were conducted using the GraphPad Prism 7.0 (GraphPad Software, San Diego, CA). The data are reported as the mean ± SD values or as representative of at least three independent experiments. *P*-values of less than 0.05 were considered significant.

## Supplementary Information

**Additional file 1.** Direct reprograming: neuronal conversion efficiency and neuronal purity.

## Data Availability

Data and material are available under request.

## Abbreviations

AASDHPPT: L-aminoadipate-semialdehyde dehydrogenase-phosphopantetheinyl transferase
AASS: Alpha-aminoadipic semialdehyde synthase
ACP: Acyl carrier protein
Aft1: Activator of iron transcription protein 1
Aft2: Activator of iron transcription protein 2
ALDH1L1: Cytosolic 10-FTHFDH
ALDH1L2: Mitocondrial 10-FTHFDH
ANOVA: Analysis of variance
CoA: Coenzyme A
CoPAN: CoA synthase protein-associated neurodegeneration
DAPI: 4′,6-Diamidino-2-fenilindol
DCA: Dichloroacetate
10-FTHFDH: 10-Formyltetrahydrofolate dehydrogenase
FASI: Fatty acid biosynthesis type I
FASII: Fatty acid biosynthesis type II
ISD11: Iron-sulfur protein biogenesis, desulfurase-interacting protein 11
KDH: Alpha-ketoglutarate
LYRM4: LYR Motif Containing 4
MAP2: Microtubule associated protein 2
MePAN: Mitochondrial enoyl CoA reductase protein-associated neurodegeneration
mtACP: Mitochondrial ACP
MTND1: NADH-ubiquinone oxidoreductase chain 1
NBIA: Neurodegeneration with brain iron accumulation
NDUFA9: NADH dehydrogenase (ubiquinone) 1 alpha subcomplex subunit 9
NFS1: NFS1 cysteine desulfurase
PDH: Pyruvate dehydrogenase
PDH-E2: Pyruvate dehydrogenase-E2
PKAN: Pantothenate kinase-associated neurodegeneration
PANK2: Pantothenate kinase 2
PPTase: Phosphopantetheinyl transferase
REST: RE1-silencing transcription factor
VPA: Valproic acid sodium salt
