# Supplementary material for: Down regulation of the expression of mitochondrial phosphopantetheinyl-proteins in pantothenate kinase-associated neurodegeneration: pathophysiological consequences and therapeutic perspectives
Source: Orphanet J Rare Dis. 2021 May 5;16:201. doi: 10.1186/s13023-021-01823-3 (PMC8101147; doi:10.1186/s13023-021-01823-3)
Supplement: Supplementary file 1 — Additional file 1. Direct reprograming: neuronal conversion efficiency and neuronal purity. [file 13023_2021_1823_MOESM1_ESM.pdf]

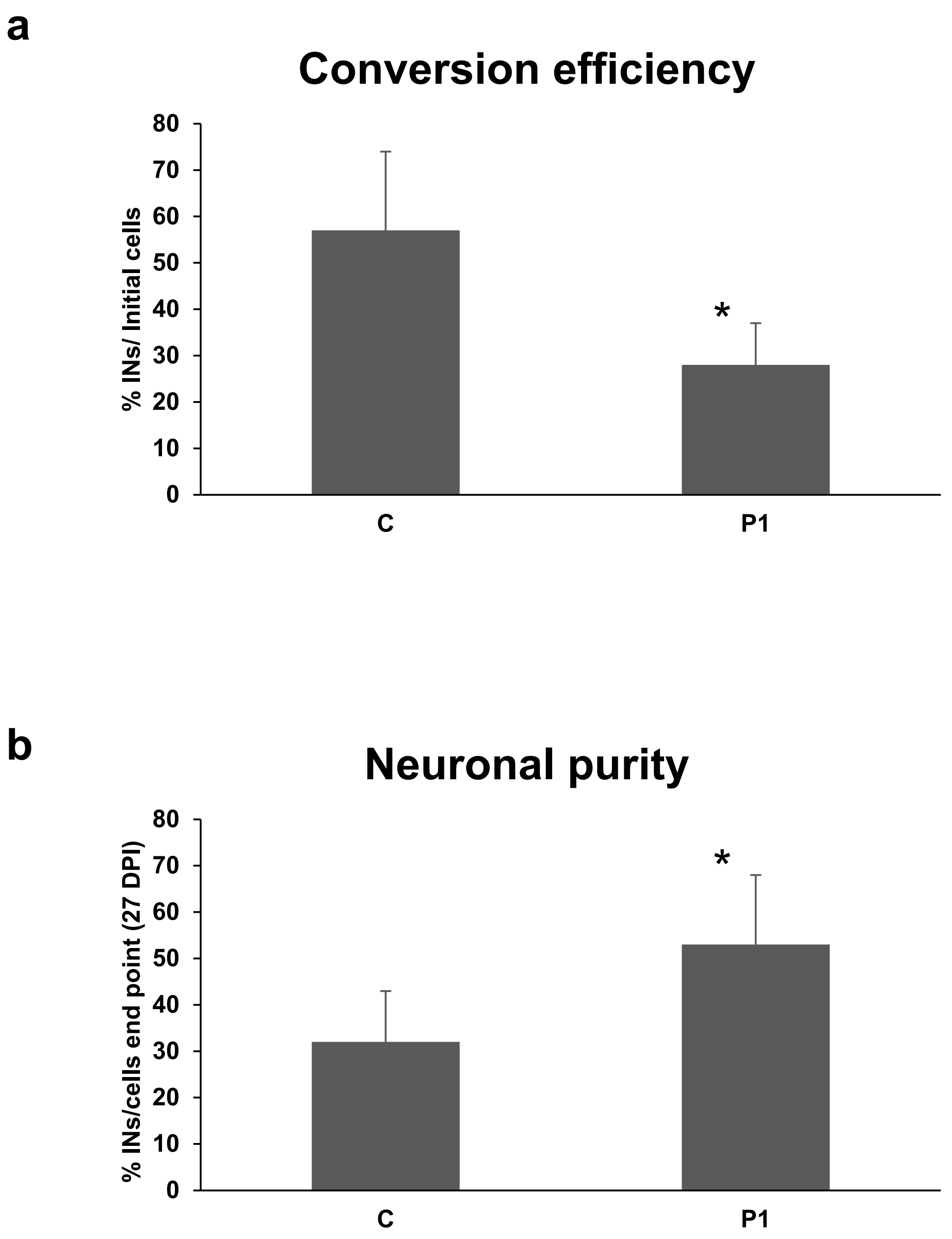

Supplementary Fig. S1. Tau/MAP2 immunostaining was used to calculate the conversion efficiency and the neuronal purity, and the results are plotted in panels **a and b**. The data are shown in the plots as the mean±SD of three independent experiments (at least 50 neurons for each condition and experiment were examined), $^*p<0.05$ between control and PKAN P1fibroblasts.
